# Can a Healthy Lifestyle Prevent Disability Pension among Female Healthcare Workers with Good and Poor Self-Rated Health? Prospective Cohort Study with 11-Year Register Follow-Up

**DOI:** 10.3390/ijerph191710631

**Published:** 2022-08-26

**Authors:** Álvaro Morera, Joaquín Calatayud, Rubén López-Bueno, José Casaña, Jonas Vinstrup, Rúni Bláfoss, Thomas Clausen, Lars Louis Andersen

**Affiliations:** 1 Exercise Intervention for Health Research Group (EXINH-RG), Department of Physiotherapy, University of Valencia, 46010 Valencia, Spain; 2 National Research Centre for the Working Environment, 2100 Copenhagen, Denmark; 3 Department of Physical Medicine and Nursing, University of Zaragoza, 50009 Zaragoza, Spain; 4 Research Unit for Muscle Physiology and Biomechanics, Department of Sports Science and Clinical Biomechanics, University of Southern Denmark, 5230 Odense, Denmark; 5 Department of Health Science and Technology, Aalborg University, 9220 Aalborg, Denmark

**Keywords:** disability pensions, healthcare workers, smoking, physical activity, BMI

## Abstract

Background: Our purpose was to investigate whether healthy lifestyle habits prevent disability pension among female healthcare workers. Methods: We conducted a prospective cohort study with an 11-year register follow-up in which 8159 female healthcare workers from Denmark completed a questionnaire concerning self-rated health, work environment, leisure-time physical activity (LTPA), smoking, and body mass index (BMI). Data on disability benefit payments were obtained from the Danish Register for Evaluation of Marginalization during an 11-year follow-up. Potential confounders included age, occupational education, psychosocial work factors, and physical exertion during work. Results: Among workers in good health at baseline, smoking, obesity, and low levels of LTPA were risk factors for disability pension during 11-year follow-up. Among workers with poor health, only low levels of physical activity were a risk factor for disability pension. Conclusions: This underscores the importance of a healthy lifestyle, specially being physically active, for preventing premature exit from the labor market in female healthcare workers.

## 1. Introduction

In most European countries, life expectancy has increased during the previous decades [1]. For that reason, many governments have increased the legal retirement age, with the aim of increasing the proportion of active workers relative to retirees [2,3]. Therefore, maintaining the good health of the ageing workforce is of vital importance in order to maintain a productive working life. Despite a general improvement in the quality of life in European countries, the number of individual disability pensions is increasing [4]. In 2018, 1712 billion was spent across European pensions, representing 12.7% of the total economic output, and more than a quarter of the European population (26.5%) received a pension this year [5]. Being unable to work until statutory pension age has adverse consequences for individuals and society, where it is not only related with a burden of the national economy but also a variety of negative relational and psychological outcomes [3,6]. For these reasons, preventing premature exit from the labor market in terms of disability pensions is a major aspect of interest.

While the incidence of disability retirement is reported throughout all areas of work, the highest is among workers in the health care sector [7]. Some prospective cohort studies showed how nursing professionals and care assistants had a higher risk of a disability pension compared to other occupations [7,8]. These findings might be related to the high mental and physical demands of their job, including a high risk of suffering musculoskeletal disorders [9]. In fact, mental and musculoskeletal disorders are the most usual causes of a disability pension [10,11]. The majority of these physical demands consisted of lifting or carrying heavy loads, working in awkward postures, and repetitive movements, while the psychologically stressful conditions included time pressure, low job control, little social or supervisor support, effort-reward imbalance, or work-life conflict [9,12]. However, different modifiable lifestyle factors, such as a low level of education [13], physical illnesses [13], and alcohol consumption [14], have been associated with a disability pension, having an important role on it. For instance, in Finland, mental and behavioral disorders exceeded musculoskeletal disorders as the principal reason of a disability pension in 2019 [15]. Within lifestyle factors, smoking, physical inactivity, and high body-mass index (BMI) are associated with higher sickness absence rates, as shown by a multicohort study from the UK, France, and Finland [16], as well as in another prospective cohort study conducted in Denmark with female healthcare workers [17]. Nonetheless, the association between these lifestyle factors and a disability pension remains poorly understood due to their complexity and due to limited evidence [18]. In addition, no previous studies have examined the relationship of these variables among healthcare workers with good and poor self-rated health. Establishing the relationship between these factors would help to identify workers with a greater risk of a disability pension and thus be able to develop successful interventions to reduce premature exit from the labor market and to promote appropriate health behaviors.

Therefore, the aim of the study was to determine the importance of a healthy lifestyle to prevent a disability pension among female healthcare workers with good and poor self-reported health. The research question was: Is there a difference between having healthy or unhealthy lifestyle habits for being granted with a disability pension among female healthcare workers with good and poor self-reported health?

We hypothesized that healthy lifestyle habits and specifically low rates of smoking and obesity and high levels of leisure-time physical activity (LTPA) would be associated with a decreased risk of a disability pension in healthcare workers with good and poor health.

## 2. Materials and Methods

### 2.1. Study Design and Population

This prospective cohort study with an 11-year register follow-up follows the STROBE guidelines [19]. Baseline information was collected between late 2004 and spring of 2005. The questionnaire was sent to 12,744 healthcare workers in eldercare in Denmark, obtaining 9949 responses (78%). Of the total sample, male respondents (n = 234) and workers who were not directly engaged in care services (n = 1021 of which some were also included in the male population of 234) were excluded from the present analyses. Therefore, the final sample of participants in this study was 8159 female healthcare workers in eldercare.

The Danish Data Protection Agency was notified of and registered the study. According to Danish law, questionnaire- and register-based studies need neither approval from ethical and scientific committees nor informed consent. All data was de-identified and analyzed anonymously.

### 2.2. Outcome

Data on disability benefit payments were obtained from the Danish Register for Evaluation of Marginalization (DREAM) [20]. Disability benefit payments can be obtained when the municipality decides that the worker has permanent full or partial loss of work ability, following a process that involves work-, health-, education-, and social-departments. Depending on the degree of work ability, an individual can receive either a full disability pension (this implies a complete cease of the workforce) or disability benefits (this entails partial work or modification of the working conditions). For the present study, a ‘disability pension’ was defined as any type of disability benefit as a result of permanent full or partial loss of work ability, including flex or sheltered jobs, and a full disability pension, involving a total of 13 categories of disability benefits payment in the DREAM register [17].

### 2.3. Lifestyle Factors and Self-Rated Physical Activity

Self-reported level of LTPA was assessed with a single question: *‘Which description most accurately reflects your pattern of leisure-time physical activity within the previous 12 months?’*. Respondents chose one of the following response categories (ranging from low to high duration and intensity): (i) Mainly sedentary or light physical activity for less than 2 h per week (e.g., reading, watching television, going to the cinema); (ii) Light physical activity for 2–4 h per week (e.g., going for a walk, light gardening, light physical exercise); (iii) Light physical activity for more than 4 h per week or vigorous physical exercise for 2–4 h per week (e.g., fast jogging or cycling, heavy gardening, exercise where you are sweating and breathing heavily); and (iv) vigorous physical exercise for more than 4 h per week or regular training/competitions several times per week [21]. Subsequently, categories i and ii were merged and defined as ‘low physical activity’, category iii as ‘moderate physical activity’, and category iv as ‘high physical activity’.

Smoking was defined from the question *‘Do you smoke?’*, with the response categories *‘yes’*, *‘I did smoke, but I quit’*, *‘I have never smoked’*. The first category was defined as ‘smoker’ and the latter two merged into ‘non-smoker’.

BMI (kg/m^2^) was determined from the self-reported height and weight of the respondents [BMI = weight (kg)/ height^2^ (m^2^)]. Consequently, BMI was categorized according to the World Health Organization classification of underweight (<18.50), normal range (18.50–24.99), overweight (25–29.99), and obese (>30.00).

Individual self-rated health of each participant was measured with a 5-point Likert scale (from poor to excellent) with the question: *How would you say your overall health is?* This method demonstrated good validity and reliability for measurement of general health [22].

### 2.4. Confounders

Potential confounders from the baseline questionnaire included age (continuous variable), occupational education (categories of specific healthcare education, e.g., social and health care assistant, social and health care helper, nurse, nurse aide, therapist, none), psychosocial work factors (emotional demands, influence at work, role conflicts, quality of leadership), and physical exertion during work (7-point scale from very, very light to very, very strenuous), as described elsewhere [3].

### 2.5. Statistical Analyses

Using the Cox proportional hazards model (PHREG procedure of SAS 9.4, SAS Institute, Cary, NC, USA) we estimated hazard ratios (HR) and 95% confidence intervals (95% CI) for a disability pension. Participants were followed up for 11 years in the DREAM register or until censoring, which occurred for voluntary early retirement pension, state pension, emigration, or death. When an individual had a registered disability benefit payment or died in any given week within the follow-up period, the survival times were non-censored and referred to as event times. Multiple imputations replaced missing values. The predictor variables were the respective lifestyle variables (mutually adjusted). The analyses were controlled for the confounders described above. *p*-Values below an alpha-level of 0.05 were considered as statistically significant differences.

## 3. Results

Table 1 shows the self-rated health of the 8159 female healthcare workers in eldercare, where 7215 workers reported having good (good, very good, or excellent) self-rated health and 944 poor health (poor or less good). During the 11-year follow-up, 937 (11.5%) of the total population were granted a disability pension, whereas this was the case for 604 (8.4%) of these reporting good self-rated health and 333 (35.3%) of those reporting poor self-rated health at baseline.

Table 2 describes the baseline characteristics and lifestyle factors of the female healthcare workers in eldercare. The mean age of the study population was 44.9 years for the good self-rated health group and 47.9 years for the poor self-rated health group. Among the good self-rated health group, approximately one third of the participants were non-smokers. The distribution of the BMI was: 2% were underweight, 58.1% had normal weight, 28.4% had overweight, and 11.5% were obese. For the LTPA, 45.1% of the workers had a low level of physical activity, 50.1% were moderately active, and only 4.8% were highly active. Among the workers with poor self-rated health, similar to the other group, one third of the participants were non-smokers. BMI was distributed as follows: 2.2% were underweight, 48.5% had normal weight, 32.3% had overweight, and 16.7% were obese.

Table 3 illustrates the age-stratified analyses with hazard ratios for a disability pension during the 11-year follow-up among female healthcare workers in eldercare with good and poor self-rated health individually. A positive association was observed between smokers with good self-reported health and a disability pension (Hazard ratio = 1.64; 95% CI 1.39–1.93), however the poor self-rated health group showed no significant effect.

In relation to BMI, we found no interaction with self-rated health for the risk of a disability pension. However, stratified analyses showed that obesity among those with good self-rated health increased the risk of a disability pension (Hazard ratio = 1.53; 95% CI 1.20–1.93).

With regard to LTPA, we only observed an association between low levels of LTPA (moderate levels as reference) and an increased risk of a disability pension among female healthcare workers in eldercare with good and poor self-rated health (Hazard ratio = 1.36; 95% CI 1.15–1.60; Hazard ratio = 1.33; 95% CI 1.04–1.70). In addition, being highly physically active does not confer additional protection.

## 4. Discussion

The main findings of the present study are that healthy lifestyle factors (BMI, smoking, and especially LTPA) have a preventive role for a disability pension among female healthcare workers in eldercare. Importantly, the potential for preventing a disability pension through a healthy lifestyle is higher when workers experience good health. When workers have already developed poor health, only physical activity seems to prevent a disability pension. This underscores the importance of a healthy lifestyle, especially the benefits of physical activity, as prevention of a premature exit from the labor market in female healthcare workers in eldercare.

Our results evidenced how low levels of LTPA, increase the risk of a disability pension during an 11-year register follow-up among female healthcare workers in eldercare. Besides, we observed no additional protection with vigorous LTPA compared to the moderately active group, and no significant interaction was found between self-rated health and LTPA. Therefore, increasing LTPA to at least a moderate level among all healthcare workers in eldercare, both those with poor and good self-rated health, could be important to prevent a disability pension. In accordance with our findings, a study among 55-year-old public-sector workers [15] concluded that a moderate level of LTPA reduced the risk of sickness absence and a disability pension, and no added protection was found for vigorous levels of LTPA. Moreover, a meta-analysis [23], including 10 studies found that lower levels of LTPA (intensity not specified) was associated with an increased risk of a disability pension. Furthermore, our results are in line with a prospective observational study with 9 years of follow-up [24] that found an inverse association between LTPA and a disability pension among all working-age men and women residing in Norway. Besides, in comparison with inactive individuals, persons who were physically active were at a 20–60% lower risk of a disability pension, depending on the intensity and duration of LTPA [24]. However, in dispute with our results, some studies [25,26] observed how decreasing LTPA from vigorous to moderate activity increased the risk of disability retirement over a follow-up of 6 years within a sample of middle-aged public sector employees. This could be explained by the different ways in which subjects were classified according to their LTPA. As well, it is likely that decreasing the amount and intensity of LTPA may be related to some underlying health problems in the long-term, which in a vicious cycle, may further reduce general physical activity, subsequently leading to a reduced physical functioning and increasing the risk of disability retirement. It could also be that workers with vigorous LTPA are a bit healthier than workers performing moderate levels of LTPA. When the “vigorous group” decrease their LTPA level to moderate, they may not be quite as healthy as before. Then, the risk of a disability pension may be a bit higher, although they may still be in good health.

In addition, in order to be able to extract more specific results, it is very important to differentiate LTPA from occupational physical activity since previous studies have shown that very high physically demanding work and exposure to lifting or carrying heavy loads increased the risk of sickness absence/disability pension combined [25]. Not in vain, higher levels of LTPA have been linked with reduced cardiovascular events and all-cause mortality risk, while higher occupational physical activity is linked with increased risks [27]. Therefore, the protective effect of being physically active during leisure time on a disability pension could be related with its associated benefits on reducing chronic disease and morbidities [28] and with preventing functional deterioration of physical capacities during ageing [29].

Our findings showed that smokers with good self-rated health were at a higher risk of a disability pension during the 11-year follow-up. Interestingly, for workers with poor self-rated health, smoking was not predictive for a disability pension, suggesting that smoking cessation alone is not sufficient in this group to prevent a disability pension. Our results are in accordance with a recent systematic review and meta-analysis [30] that revealed a positive relation between smoking and a disability pension, showing how current or former smoking women had a 23% higher risk for having a disability pension than non-smokers. A population-based twin cohort study [31] also found that current daily women smokers had an increased risk of a disability pension when compared to women who had never smoked. In contrast, a non-randomized nested pseudo-trial study [32] reported that smoking cessation may reduce the hazard ratio for a disability pension by more than 9%, compared with continuing smoking. However, this could be explained due to the fact that variables, such as the intensity and duration of smoking, were not measured, which may confound the associations between cessation and the risk of work disability. Besides, it is important to note that both men and women were chosen for the analysis, so the results could not be extrapolated to ours. Interestingly, to support the idea that no single-factor is enough to prevent a disability pension, a cohort study [33] showed that among never-smokers, ex-smokers, and moderate smokers (1–14 cigarettes per day), the practice of vigorous LTPA might help to prevent disability retirement. This was not found when performing lower levels of LTPA [33]. However, heavy smokers (15 or more cigarettes) had an increased risk of disability retirement independently of the amount and intensity of LTPA [33]. In summary, while smoking cessation alone is not sufficient to prevent a disability pension in the poor self-rated health, there is ample evidence that smoking is responsible for many diseases, such as heart disease, lung and throat cancer, lung disease, and stroke, all of which increase the risk of receiving a disability pension [34].

We found that BMI did not interact with self-rated health in the risk of a disability pension. However, stratified analyses showed that obesity among those with good self-rated health increased the risk of a disability pension. This was not the case for those with poor health, suggesting that, as occurred with smoking, reducing BMI alone is not sufficient in this group to prevent disability. Our findings support those obtained in an earlier meta-analysis of 28 longitudinal studies [23], showing that obesity and to a lesser extent overweight, are associated with future disability pension in women and men. However, it should be noted that the study did not use the normal World Health Organization cut-off values, likely overestimating the relationships. In line with our findings, different studies conducted with males also showed a significantly elevated risk of a disability pension in overweight [33] and particularly in obese subjects [35,36]. These findings have been recently corroborated in a systematic review and meta-analysis of 27 studies [37]. Moreover, the relation between BMI and all-cause disability retirement were similar in both sexes. Interestingly, low cardiorespiratory fitness is a risk factor for future disability regardless of BMI, and being moderately or highly fit mitigated the risk of a disability pension in all BMI categories [36], which, in accordance with our results, suggests that reducing BMI alone is not sufficient to prevent a disability pension. Therefore, the influence of BMI with a disability pension has to be taken into account since higher BMI has been linked with a higher incidence of cardiovascular events and chronic conditions, including type 2 diabetes, gallbladder disease, osteoarthritis, hypertension, dyslipidaemia, and coronary heart disease [38]. Summarizing all our findings, it should be emphasized that, instead of promoting separate health behaviors, promoting healthy behaviors in general during our life decreases the risk of suffering a disability pension.

### Limitations and Future Research Directions

The present study has some limitations. A limitation is that predictor and confounder variables were measured only at baseline, thus the study could not take into account possible changes over time. The utilization of self-reports to measure some variables could be a limitation because outcomes could be underestimated or overestimated by social desirability or overall bias. On the other hand, these measures are valid and reliable [22]. In addition, readers should be aware that our results could not be extrapolated to male healthcare workers. A strength of our study was the large sample size and long duration of follow-up. Importantly, disability pension data were obtained from national registers with complete follow-up measures, therefore, no selection nor attrition bias affected our outcome. Despite its limitations, our findings demonstrate that maintaining healthy lifestyle habits, especially physical activity, are really important for preventing a disability pension in female healthcare workers in eldercare. These findings have significant implications for preventing premature exit from the labor market. Future studies should evaluate the effectiveness of interventions to improve lifestyle habits on preventing a disability pension.

## 5. Conclusions

The potential for preventing a disability pension through maintaining healthy lifestyle habits (BMI, smoking, and especially LTPA) is greater for workers in good health. For workers reporting poor health, only physical activity was found to reduce risk of a disability pension. This underscores the importance of a healthy lifestyle, especially the benefits of physical activity, as prevention of premature exit from the labor market in female healthcare workers in eldercare.

## Figures and Tables

**Table 1:** Participants self-rated health and number of disability pensions.

How Would You Say Your Overall Health Is?	Disability Pension during 11-Year Follow-Up		
	No	Yes	Total
Good health %	6611	604	7215
	91.6%	8.4%	
Poor health %	611	333	944
	64.7%	35.3%	
Total	7222	937	8159
	88.5%	11.5%	

**Table 2:** Participants baseline characteristics and lifestyle factors.

	Good Health				Poor Health			
	N	Mean	SD	%	N	Mean	SD	%
**Age**	7215	44.9	10.1		944	47.9	9.0	
**Smoking**								
No	4551			63.1	570			60.4
Yes	2664			36.9	374			39.6
**Body Mass Index (kg/m^2^)**								
Underweight	142			2.0	23			2.4
Normal weight	4190			58.1	458			48.5
Overweight	2051			28.4	305			32.3
Obesity	832			11.5	158			16.7
**Physical activity level during leisure**								
Low	3253			45.1	544			57.6
Moderate	3613			50.1	369			39.1
High	349			4.8	31			3.3
**Physical exertion during work (1–7)**	7215	3.8	1.2		944	4.3	1.3	
**Psychosocial work factors (0–100)**								
Emotional demands	7215	45.4	18.2		944	52.0	19.0	
Influence at work	7215	45.7	20.3		944	39.9	21.4	
Role conflicts	7215	41.1	15.4		944	45.2	16.8	
Quality of leadership	7215	57.7	21.3		944	50.7	22.8	

**Table 3:** Health-stratified analyses of the health-related factors.

	Hazard Ratio (95% CI)		*p*-Value
	Good Health	Poor Health	Interaction
Smoking			
No	1	1	
Yes	**1.64 (1.39–1.93)**	1.15 (0.90–1.46)	**0.017**
BMI			
Underweight	1.43 (0.90–2.28)	1.38 (0.69–2.75)	
Normal weight	1	1	0.283
Overweight	1.06 (0.88–1.28)	1.07 (0.82–1.40)	
Obesity	**1.53 (1.20–1.93)**	1.04 (0.74–1.45)	
Leisure time physical activity			
Low	**1.36 (1.15–1.60)**	**1.33 (1.04–1.70)**	
Moderate	1	1	0.976
High	0.89 (0.56–1.41)	0.82 (0.38–1.75)	

Adjusted for the three lifestyle factors, age, psychosocial and physical work environment, and education. Note: only a fully adjusted model (not a minimally and fully). Bold numbers denote statistically significance.

## Data Availability

The authors encourage collaboration and use of the data by other researchers. Due to Danish data protection legislation, micro data cannot be made publicly available. Researchers interested in using the data for scientific purposes should contact the project leader Lars L. Andersen, lla@nfa.dk.
